# Enhanced microwave-assisted methodology for obtaining cutin monomers from tomato peel

**DOI:** 10.3389/fchem.2025.1734422

**Published:** 2026-01-05

**Authors:** Constanza Maciel, María José Cocero, Rafael B. Mato

**Affiliations:** 1 Research Institute on Bioeconomy - BioEcoUVa, PressTech Group, University of Valladolid, Valladolid, Spain; 2 Department of Chemical Engineering and Environmental Technology, University of Valladolid, Valladolid, Spain

**Keywords:** alkaline hydrolysis, biopolyester, cutin characterization, dihydroxyhexadecanoic acid, tomato peels

## Abstract

Tomato pomace is an abundant by-product of the agri-food industry with a peel rich in cutin, a plant polyester that can be depolymerized to monomeric building blocks to develop bio-based materials. Because of cutin’s crosslinked, three-dimensional structure, alkaline hydrolysis has typically required long reaction times (up to 24 h) to achieve complete depolymerization into its monomers, which hinders the potential development of exploitation processes. In this paper, the effect of temperature and heating mechanism (conventional versus microwave-assisted hydrolysis) on monomer production yield and final product composition of the hydrolysis process were studied. The comparison of the two methods was also based on a detailed kinetic analysis of the hydrolysis processes. The results showed that the usage of microwaves at 120 °C allowed to reduce the reaction time from 24 h at 100 °C to only 1 h, with no significant differences to the conventional hydrolysis method in terms of monomeric composition, but with higher yields. This reduction in processing time promotes the development of new applications from the corresponding monomers and facilitates cutin characterization for analytical purposes.

## 1 Introduction

Tomato is one of the five most produced and consumed crops worldwide, and processed tomatoes consumption in Europe is expected to exceed 50 million tons by 2025 (Data Tomato production FAO, 2023). However, the fraction of these residues -mainly peels and seeds- that cannot be used as a source of food, can be transformed into valuable by-products, reducing greenhouse gas emissions through decomposition or fermentation and leading to a circular economy (Fritsch et al., 2017; Guzmán-Puyol et al., 2021; Pollard et al., 2008; Rahman, 2021). The tomato peels are mainly composed of a biopolyester named cutin, which ─ depending on the harvesting conditions and the specific tomato variety ─ can reach up to 80% w/w (Benítez et al., 2004; Dominguez et al., 2011; Kolattukudy, 1980).

In nature, this biopolyester acts as a framework for the aerial part of the plants to accommodate the other elements of the cuticle (Heredia et al., 2024), which consist of polysaccharides that offer structural support to the material; epicuticular and intracuticular waxes, that prevent the water loss; and phenolic compounds, that shield the plant from UV radiation (Fich et al., 2016; Lara et al., 2019). Cutin from tomato peel is composed of more than 75% of 10,16-dihydroxyhexadecanoic acid (Marc et al., 2021; Philippe et al., 2016; Reynoud et al., 2021).

The potential of cutin for the development of bio-based materials stems from multiple attributes, including its three-dimensional structure, the self-assembly and self-esterification properties of its C16 and C18 hydroxy fatty acids, and its remarkable hydrophobicity (Domínguez et al., 2015; Escórcio et al., 2022). This unique structural feature, resulting from the crosslinking between its monomers (Bento et al., 2021; Fich et al., 2016; Kolattukudy, 2002), enables a wide range of potential applications (Tedeschi et al., 2018), not only for the biopolymer itself, but also for the oligomers or monomers obtained from it.

Currently, different approaches are being taken to valorize the cutin from this residue. Some research groups have obtained innovative materials by mixing cutin with linear polymers like poly(lactic acid) (Arrighetti et al., 2024; Merino et al., 2022) or films for food packaging by mixing with chitosan (Simões et al., 2023). Escórcio et al. obtained antimicrobial cutin-derived oligomers (Escórcio et al., 2022), Marc et al. deconstructed the cutin network to produce hydrophobic elastomers (Marc et al., 2021) and shape-memory polyesters (Marc et al., 2024), and Buratti et al. developed poly(ester-urethane) coatings (Buratti et al., 2024). Moreover, Heredia’s group illustrated how cutin can be used for packaging materials (Benítez et al., 2015; Benítez et al., 2018; Heredia-Guerrero et al., 2017; Tedeschi et al., 2018). Taken together, these cases demonstrate that, beyond the interest of its three-dimensional architecture, cutin’s monomers themselves offer a wide range of possibilities.

In all the cases mentioned above, to obtain and characterize the monomers which constitute the cutin, it is necessary to cleave the ester bonds that form the polymeric network. Several methodologies have been explored for this purpose, the most common being exhaustive alkaline hydrolysis and transesterification with methanol, reductive cleavage with LiAlH4 in tetrahydrofuran, and enzymatic methods using conventional heating (Kolattukudy, 2002). All processes described to date for this purpose involve long reaction times with multiple steps, and the use of expensive solvents and chemicals (Benítez et al., 2018; Gómez-Patiño et al., 2015; Heredia-Guerrero et al., 2017; Kolattukudy, 2002; Marc et al., 2021). Table 1 compiles prior studies on alkaline depolymerization of cutin (NaOH or KOH), where operating conditions vary widely. Unfortunately, the extent of hydrolysis is not monitored in some of them. Most reports employ prolonged reaction times (typically >4 h) and, in several cases, alcoholic co-solvents such as methanol or ethanol to accelerate depolymerization. In contrast, our objective is to develop an efficient alkaline hydrolysis protocol that operates with short residence times, uses only aqueous sodium hydroxide (no co-solvents), and enables immediate analysis of the product stream. This approach reduces processing steps, minimizes solvent-related hazards and waste, and is designed to yield monomeric products suitable for subsequent materials applications without compromising composition or overall yield.

**TABLE 1:** Alkaline hydrolysis under conventional heating reported in literature.

Solvent	Temperature	Time	Solid ratio	References
NaOCH_3_ 0.1M in methanol	Reflux	3.5 h	1% w/v	Graça and Lamosa (2010)
NaOH 0.75M	65 °C–130 °C	<6 h	3% w/v	Cigognini et al. (2015)
NaOH 1 M	100 °C	24 h	2% w/v	Benítez et al. (2018)
KOH in ethanol 95%	Room T	24 h	5% w/v	Marc et al. (2021)
NaOH 0.5 in methanol/water	95 °C	4 h	​	Bento et al. (2021), Escórcio et al. (2022)
KOH in ethanol 95%	95 °C	4 h	5% w/v	Simões et al. (2023)

Microwave-assisted hydrolysis have been successfully used in many biomass valorization processes, including, among many others: biomass conversion into fuels or value-added chemicals (Asomaning et al., 2018), hydrothermal treatment for biomass valorization (Gao et al., 2021), optimization of acid hydrolysis of polysaccharides for monosaccharide composition analysis (He et al., 2018), extracting water-soluble components from tea leaves residues and obtaining cutin in its polymeric form as a residue after processing (Tsubaki et al., 2008). In residues from the tomato industry, microwaves have been used to efficiently recover by-products such as pectins, carotenoids, and antioxidants (Aldana-Heredia et al., 2024; Golub et al., 2025; Solaberrieta et al., 2022). Nevertheless, despite the promising application of microwave technology in biomass processing, there is no previous reported work using this methodology on tomato residues to obtain cutin monomers.

Microwave irradiation produces heating by various mechanisms, the most relevant in this case being: (1) delayed molecular motion, caused by the dipolar rotation of water molecules as they follow the fluctuations of the electric field, and (2) ionic conduction, caused by the generated electromagnetic field (Quitain et al., 2017). In contrast to other methods, microwave irradiation produces efficient internal heating through direct coupling of microwave energy with the molecules involved in the reaction mixture, which can lead to a reduced reaction time (Kappe and Stadler, 2005; Quitain et al., 2022; Tsubaki et al., 2013) and less heat losses (Cantero et al., 2019). Previous studies showed that hydrothermal hydrolysis was improved with the use of salts compared to conventional heating without the addition of electrolytes (Tsubaki et al., 2012; Tsubaki et al., 2016).

The objective of this work is to develop a novel process for producing monomers using microwave-assisted alkaline hydrolysis, with a reduced operation time compared to conventional methods reported in the literature and without the use of co-solvents. The results obtained with this approach will be compared to those of the conventional alkaline aqueous hydrolysis method in terms of product composition, hydrolysis rate, and overall yield. This process also enables faster characterization of the composition of the monomers obtained from cutin.

## 2 Materials and methods

### 2.1 Chemicals

Chloroform ≥99% for HPLC stabilized with ethanol from Thermofisher (Massachusetts, United States). Methanol 99.8% for HPLC from Fisher Chemical (Pittsburgh, United States). Hydrochloric acid 37%, sodium hydroxide pellets 98% and toluene analytical grade from PanReac (Castellar del Vallès, Spain). Pyridine anhydrous 99.8%, N,O-Bis(trimethylsilyl)trifluoro-acetamide ≥99% and hexadecane analytical grade from SigmaAldrich (Missouri, United States). Water used was Milli-Q grade obtained with an equipment from Millipore (Milan, Italy).

### 2.2 Plant material

Tomato pomace was supplied by Pronat Company (Don Benito, Badajoz, Spain), as a residue from processing 300,000 tons of tomatoes per campaign (5,600 tons per day)^1^. This pomace consists in between 10% and 20% (w/w) of peels, being seeds and fibers the rest of it. The tomato peel fraction was the only concern of this work because of its high content of cutin. A detailed characterization of this pomace can be found in previous work from our research team (Leontijevic et al., 2025). Upon receipt, the biomass was processed by aqueous decantation in a water tank. Gentle manual stirring promoted separation: the peel-rich fraction floated and was recovered, whereas seeds and fibrous residues settled and were removed. They were then dried in an oven at 50 °C for about 5 days. The dried peel fraction was first grinded with a kitchen blender and then milled in a Retsch PM100 ball miller (Haan, Germany) for 4 h (cycles of 1 min milling and 14 min cooling break), using a program of 450 rpm. The tomato peel powder (TP) obtained from this process was stored at room temperature for subsequent assays.

**FIGURE 1:**
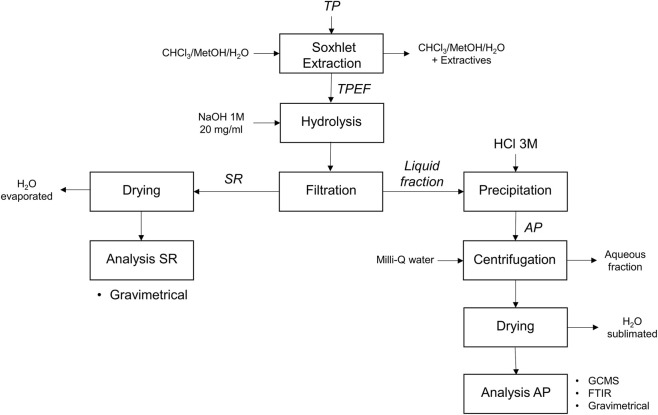
Hydrolysis process diagram (BHMW and BHSB).

The experimental procedure for the subsequent hydrolysis process is outlined in Figure 1, showing the steps and analyses involved in the process.

### 2.3 Extractives removal

To facilitate the hydrolysis, the cascade method with Soxhlet extraction (Sluiter et al., 2008) was used as a pre-treatment for removing the extracts (waxes and unbonded compounds) from the biomass, which represented around 13% of the composition (Benítez et al., 2018). It involved three steps: (1) 5 h using chloroform to remove waxes, (2) 5 h with methanol to remove unbounded phenolics, and (3) 24 h with water to remove sugars, as reported in previous work (Leontijevic et al., 2025). Approximately 5 g of TP (*m*_*TP*_ in Equations 1 and 2) and 190 mL of each solvent were used. Thereafter, the tomato peel extract free (TPEF) was dried overnight in an oven at 50 °C and then manually grinded again to get a uniform powder.

### 2.4 Conventional alkaline hydrolysis in silicon bath

To obtain and characterize the monomers of cutin, this biomass must be subjected to alkaline hydrolysis, which is the most effective process to cleave the ester bonds that compose it. The results obtained in these conventional alkaline hydrolysis experiments will be used as a reference for comparison with those obtained through microwave alkaline hydrolysis, which are the true focus of this study. For the conventional method, a silicon bath was used to heat the samples up to 100 °C and 120 °C (Basic Hydrolysis in Silicon Bath, BHSB). BHSB was carried out in pressure tubes 15 Ace-Thred of 120 mL capacity from Ace Glass (vapor pressure is close to 2 bar), with internal magnetic stirring set at 600 rpm, immersed in an isolated silicon oil bath. The tubes were filled to 60 mL with a suspension of 0.02 g of TPEF (Tomato Peel Extractives Free) per mL of a 1M NaOH solution. After completion of the programmed reaction time, the tubes were immediately cooled down in a manually agitated cold-water bath (around 15 °C) to stop the hydrolysis process. At 100 °C, no satisfactory results were obtained due to the low hydrolysis kinetics achieved at this temperature, so the experiments with microwave-assisted hydrolysis were only performed at 120 °C. Following preliminary trials, the reaction time varied between 30 and 120 min.

### 2.5 Microwave-assisted alkaline hydrolysis

The microwave-assisted alkaline hydrolysis (Basic Hydrolysis in Microwave, BHMW) was performed using a single-mode Monowave 300 microwave oven (Anton Paar). Borosilicate glass vials G30 (Anton Paar) with 20 mL capacity and pressure resistance were used, filled with a suspension of around 0.3 g of TPEF in 15 mL of 1M NaOH, the same solid concentration used in conventional hydrolysis. A constant temperature program was selected for a given hydrolysis time. Reaction times from 10 to 120 min were tested and the temperature was set at 120 °C after the results obtained with conventional hydrolysis. Higher temperatures or OH^−^ concentrations may require more resistant and specific equipment. The chosen reaction times were evaluated by tracking reaction progress using the analyses presented below.

All experiments in Sections 2.4 and 2.5 were performed in triplicate. Gravimetric analyses (Section 2.6) were additionally done on the initial solution before starting the heating process to calculate yield values at t = 0.

### 2.6 Gravimetrical analysis

At the pH value used in the experiments, the hydrolyzed monomers remain solubilized. To isolate the remaining solid that had not been hydrolyzed (SR: solid residue), the samples obtained in the hydrolysis, once cooled, were vacuum filtered at room temperature using filter paper, and washed thoroughly with Milli-Q water. This procedure was carried out in both hydrolysis methods (see Figure 1). The SR was then dried overnight at 50 °C and gravimetrically quantified after no meaningful changes between the measurements (*m*_*SR*_). The filtrated liquid containing the monomeric solution was acidified at room temperature with a 3M HCl solution until pH 3 to precipitate the acid monomers (AP: acid precipitate), as it was done in earlier work (Benítez et al., 2018). This AP was separated from the aqueous fraction by centrifugation at 9,000 rpm for 30 min at 20 °C, followed by three washing steps with Milli-Q water, each including centrifugation for 10 min at 20 °C. The aqueous fraction consists of a NaCl solution formed by the acidification of the samples after hydrolysis, some phenolic compounds, and some soluble monomers and water from the washing. A liquid-liquid extraction with ethyl acetate was done to this fraction in order to recover monomers, but it was discarded owing to poor yields and extended processing times. Finally, AP was freeze dried to remove the water and quantified gravimetrically (*m*_*AP*_). An analytical balance Ohaus Adventurer Pro AV264C (260 g max, ±0.0001 g) was used for all weightings, and each experiment was performed in triplicate.

The hydrolysis yields for the monomeric acid precipitate (*Yield*_*AP*_ in Equation 1) and the non-hydrolysable residue (*Yield*_*SR*_ in Equation 2) were referred to the raw TP:

$${Yield\;}_{AP}=\frac{m_{AP}}{m_{TP}}\times 100$$ (1)

$${Yield\;}_{SR}=\frac{m_{SR}}{m_{TP}}\times 100$$ (2)

Where *m*_*TP*_ is the initial mass of TP before the Soxhlet extraction, *m*_*AP*_ the mass of monomeric acid precipitate, and *m*_*SR*_ the mass of non-hydrolyzed solid residue after the hydrolysis.

### 2.7 FT-IR analysis

The Fourier transform infrared spectroscopy (FTIR) was used for characterization of the dried hydrolyzed samples (AP) and was performed using a Bruker Tensor 27 equipped with a universal attenuated total reflectance accessory with internal diamond crystal lens. Spectra were recorded in the wavelength range from 4,000 cm^−1^ to 400 cm^−1^ with a resolution of 4 cm^−1^ and 64 scans. The recorded spectra were baseline-subtracted and normalized to the maximum peak intensity.

### 2.8 GC-MS analysis

Gas chromatography-mass spectrometry (GC-MS) was performed for analysis of the monomers composition present in the acid precipitate (AP) using an Agilent 7890 GC and 5977B quadrupole MS with HP-5MS column, 30 m, 0.25 mm × 0.25 µm. For the sample preparation, a modified version of the method employed by Benítez et al. was used (Benítez et al., 2018). Around 5 mg of each sample were dissolved in 2 mL chloroform:methanol (3:1, v/v), sonicated for 20 min and then passed through 0.22 mm syringe filters. After solvent evaporation in a fume hood, 250 µL of bis(trimethylsilyl)trifluoroacetamide (BSTFA) dissolved in 50 µL of pyridine was added. An internal standard (50 µL of hexadecane in toluene, 10 µL per 25 mL) was then added to assess instrumental repeatability. Samples were heated at 70 °C for 2 h to complete derivatization prior to GC injection.

The derivatized samples were analyzed using the following temperature program: 80 °C, 2 °C min^−1^ until 116 °C, 5 °C min^−1^ until 176 °C, 2 °C min^−1^ until 270 °C, 20 °C min^−1^ until 310 °C and then hold at 310 °C for 5 min 1 μL of sample was injected at 250 °C using split-less mode, and Helium was used as a carrier gas at a flow of 1 mL/min.

Data were acquired using Mass Hunter Work Station Software B.07.00 and compounds were identified based on EI-MS (EI+, 70 eV) fragmentation patterns using the Wiley-NIST reference library and comparison with the literature.

The composition of cutin monomers was calculated as a relative abundance by the ratio between the values of the integrated peak area of each monomer and the total integrated peak area of the main peaks in the chromatogram.

The monomeric abundance is defined by Equation 3

$$monomers\;abundance\;\%=\frac{{pm}_{x}}{{pm}_{sT}}\times 100$$ (3)

Where pm_x_ is the area of the given monomer peak and pm_sT_ is the sum of the areas of the main peaks.

### 2.9 Statistical analysis

The data were analyzed by analysis of variance (ANOVA) to determine statistical differences between the methods in yields and monomer composition. Significance was defined at p < 0.05 using Statgraphics Centurion 19 (Statgraphics Technologies, Inc., United States).

## 3 Results and discussion

### 3.1 Hydrolyzed fraction

**FIGURE 2:**
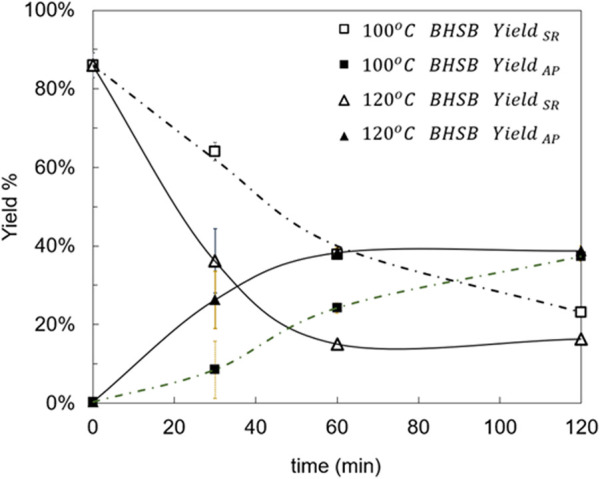
Hydrolysis yields (Yield_SR_ and Yield_AP_) at 100 °C and 120 °C with the BHSB method. Trend lines are shown for easy comparison between temperatures.

Conventional BHSB experiments were conducted at 100 °C and 120 °C, as described in Section 2.4. The evolution of the solid-residue yield (Yield_SR_) as a function of temperature and reaction time is presented in Figure 2. At ambient temperature and t = 0, the solid-residue yield is 87.1%. This 12.9% (±2.6%) reduction is due to the extractives removal described in Section 2.3 and displayed in Supplementary Table 1S in the Supplementary Material. Figure 2 shows a marked effect of temperature on the hydrolysis rate, as expected. At 120 °C, the fraction of non-hydrolyzed solid residue (SR) decreased rapidly, reaching a minimum after 60 min, and remained essentially constant thereafter at around 17%. In contrast, at 100 °C the reaction progressed more slowly, which is expected due to the lower temperature. Hydrolysis had not reached completion after 120 min, as evidenced by the higher residual SR yield at that time. A similar behavior was observed for the monomers AP yield. At 120 °C, the AP yield reached a maximum of 40% after 60 min and remained stable thereafter. At 100 °C, no clear maximum in AP yield was observed within 120 min, consistent with slower kinetics at lower temperature.

Extending the reaction beyond 60 min at 120 °C afforded only marginal additional conversion; the difference between the response at 60 min and at 120 min was as low as 2%. From a process standpoint, prolonging the reaction past 60 min is not justified, given the limited gain relative to the added time and energy consumption. According to literature, the non-hydrolyzable solid residue remaining after hydrolysis completion may contain polysaccharides such as cellulose, hemicelluloses, and pectins (Benítez et al., 2018; López-Casado et al., 2007). At shorter reaction times, it is also plausible that oligomers and cutin-derived polymeric structures persist without hydrolysis and remain in the solid fraction.

Considering the previous results, operation at 100 °C was not considered in the microwave experiments (BHMW), focusing instead on microwave hydrolysis at 120 °C. As the BHMW results showed a faster response than those of the conventional BHSH method, shorter times of 10 and 20 min were also tested in this case, to determine the kinetics in this interval more accurately.

**FIGURE 3:**
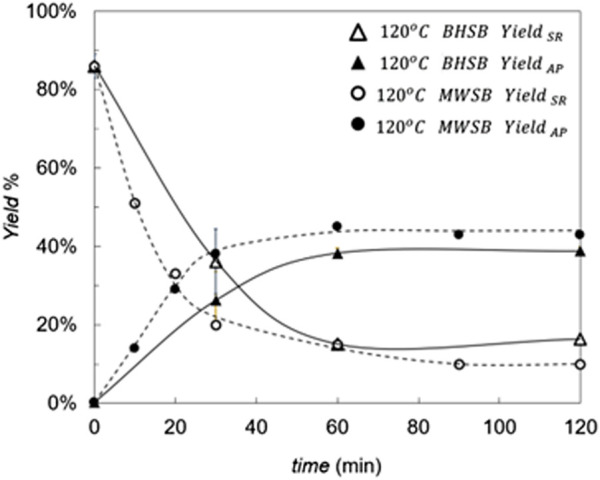
Hydrolysis yields (Yield_SR_ and Yield_AP_) at 120 °C with BHSB and BHMW methods. Trend lines are shown for easy comparison between BHSB (continuous) and BHMW (dashed) methods.

In the comparison of hydrolysis yields evolution at 120 °C between BHSB and BHMW experiments (Figure 3), it stands out that the use of microwave irradiation (BHMW) produced approximately 5% higher monomers AP yield and 5% less solid residue SR than conventional heating (BHSB) after 120 min of hydrolysis, when both processes seem to be significantly completed. The obtention of AP monomers remains stable after 40 min of reaction with BHMW, and after 60 min of reaction with conventional BHSB. In addition, initial reaction rates are higher when using microwaves, as inferred from the higher slope of both curves (SR and AP) at initial times, particularly between 0 and 30 min.

These results are consistent with the almost instantaneous volumetric heating of microwave irradiation, combined with effective stirring, which together accelerate the hydrolysis relative to conventional heating. Adequate agitation is essential to minimize thermal gradients and mitigate hot spots inherent to microwave heating. The resulting rapid and uniform temperature rise likely promotes depolymerization of cutin at short reaction times, even though microwaves themselves are unlikely to cleave chemical bonds directly (Kappe and Stadler, 2005).

A two-way multifactorial ANOVA was applied to evaluate the effect of reaction time (6 levels) and method (BHSB vs. BHMW) on yield (%SR) at 120 °C (n = 3 per condition). Both time (p < 0.001) and method (p = 0.008) had a significant effect on %SR, where microwave irradiation presented better yields in every condition evaluated. The residuals met the assumptions of normality and homogeneity of variance. Consistent with these results, previous studies on microwave-assisted processing of tomato industry by-products have also reported improved recovery of other compounds such as pectins, carotenoids, and antioxidants (Aldana-Heredia et al., 2024; Golub et al., 2025; Solaberrieta et al., 2022).

Since neither the hydrolysis yield nor the monomer-acid profile changed significantly after 1 h, this reaction time was finally chosen as the recommended one to consider the hydrolysis process completed. Conversely, if the objective of our process were partial hydrolysis of cutin to obtain oligomer mixtures, shorter reaction times of less than 30 min should be explored in BHMW. It is worth noting that not only could molecules of higher molecular weight can be found, but also aromatic compounds are likely to be present in higher relative abundance, as can be seen in the characterization Sections 3.2 and 3.3.

### 3.2 FT-IR hydrolysate characterization

**FIGURE 4:**
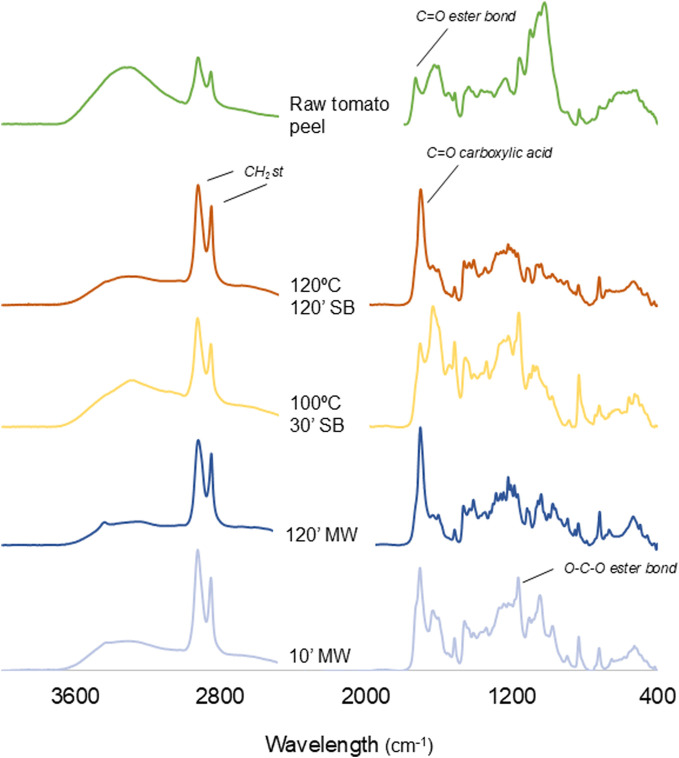
FT-IR spectra of raw tomato peel and for hydrolyzed products using mildest and harshest conditions in both methods.

The analyses of FT-IR (Figure 4; see Supplementary Table S2 in the Supplementary Material) showed in the raw TP the fingerprint of cutin esters at 1,730 cm^−1^, assigned to the carbonyl stretching of C=O in an ester bond. After hydrolysis, this band is absent and a new band appears at 1,705 cm^−1^, attributable to the C=O stretching of carboxylic acids. This spectral shift evidences cleavage of ester linkages and the formation of free carboxylic acid groups.

After 2 h of hydrolysis at 120 °C, and using both methods, we can still find some ester bonds as indicated by the presence of peaks at 1,167 and 1,108 cm^−1^ that can be assigned to the stretching from the ester bond COO-. The peak at 1,050 cm^−1^, which decreases its intensity with the reaction time, may also correspond to these bonds (Hoffman, 2005). The hypothesis that the structure of cutin is hyperbranched is consistent with these observations, as the difficulty in fully hydrolyzing its ester bonds may be due to its complex and intricate molecular arrangement.

The broad band around 3,300 cm^−1^ is assigned to the stretching vibration of OH groups interacting by H bonding. Although the main contribution to this band is expected to be due to the polysaccharides, the non-esterified OH groups from cutin will also contribute as well (Heredia-Guerrero et al., 2014). The strong bands at 2,925 and 2,850 cm^−1^, from the asymmetrical and symmetrical stretching vibration of CH_2_ groups, correspond to the aliphatic chains of the cutin monomers. The bands at 1,634, 1,515, 1,438 and 1,410 cm^−1^ indicate the presence of phenolic compounds.

### 3.3 GC-MS identification of the main monomers

**FIGURE 5:**
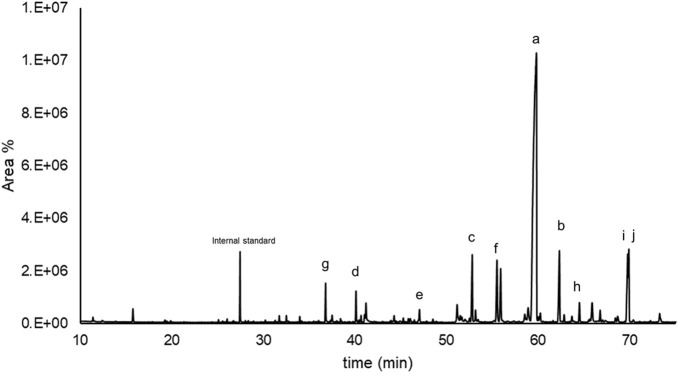
Representative GC-MS chromatogram from the monomeric composition of cutin obtained after alkaline hydrolysis of tomato peel. The letters indicate the main monomers of cutin; *a*: 10,16-dihydroxyhexadecanoic acid; *b*: 9,10,16-dihydroxyhexadecanoic acid; *c*: 16-hydroxyhexadecanoic acid; *d*: hexadecanoic acid; *e*: octadecanoic acid; *f*: hexadecanedioic acid; *g*: 4-hydroxycinnamic acid; *h*: resveratrol; *i*: naringenin; *j*: 2-hydroxy-4-(methylsulfonyl)isophthalic acid.

The main cutin monomers were identified by GC–MS analysis, and a representative chromatogram is shown in Figure 5. Minor components are not identified in the chromatogram because they are not related to cutin monomers. These minor signals likely arise from trace constituents of residual seed material, as manual separation of peels and seeds may not have been fully exhaustive. Since this study focuses on the major cutin-derived monomers, these minor non-cutin compounds were excluded from the analysis.

**FIGURE 6:**
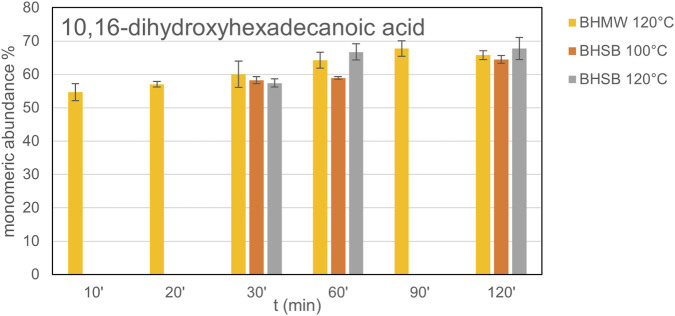
Evolution of the main monomer of cutin (10,16-dihydroxyhexadecanoic acid) as % of abundance in hydrolysates.

Their monomeric abundance percentages, as defined in Equation 3, are presented in Tables 2 and 3. Consistent with prior reports cited in the Introduction section, the predominant monomer produced by both BHMW (Table 2) and BHSB (Table 3) across all tested reaction times and temperatures is 10,16-dihydroxyhexadecanoic acid (10,16OHC16). This outcome is expected for tomato peels at an advanced ripening stage, where cutin composition is enriched in 10,16OHC16 (Philippe et al., 2016). Depending on the given reaction time, hydrolysis method and temperature, values in the range of 54%–68% of monomeric abundance were obtained, as shown in Figure 6 (data in Supplementary Table S7).

**TABLE 2:** GC-MS results from BHMW at 120 °C expressed as monomer abundance%.

Monomers	Peak*	RT	10′	20′	30′	60′	90′	120′
Acids								
Hexadecanoic acid	d	40.1	1.05 ± 0.21	1.16 ± 0.06	0.85 ± 0.14	0.84 ± 0.11	0.84 ± 0.03	0.85 ± 0.10
Octadecanoic acid	e	47.0	0.65 ± 0.06	0.51 ± 0.10	n.d.	n.d.	0.36 ± 0.01	0.35 ± 0.07
Diacids								
Hexadecanedioic acid	f	55.9	5.47 ± 2.04	4.78 ± 0.28	4.22 ± 0.58	3.85 ± 0.28	2.74 ± 0.02	2.45 ± 0.07
Hydroxy acids								
16-Hydroxyhexadecanoic acid	c	52.9	3.77 ± 1.32	4.28 ± 0.47	3.97 ± 1.14	3.35 ± 0.74	3.50 ± 0.13	3.65 ± 0.02
10,16-dihydroxyhexadecanoic acid	a	60.1	54.65 ± 2.56	57.02 ± 0.84	60.01 ± 3.97	64.25 ± 2.38	67.73 ± 2.36	65.76 ± 1.33
9,10,16-trihydroxyhexadecanoic acid	b	62.4	5.19 ± 0.68	5.76 ± 0.80	6.68 ± 0.69	8.50 ± 0.26	9.07 ± 0.17	10.25 ± 0.92
Phenolics								
4-Hydroxycinnamic acid	g	36.8	1.34 ± 0.22	1.40 ± 0.98	1.43 ± 0.58	5.04 ± 0.45	3.03 ± 1.08	3.86 ± 1.03
Resveratrol	h	64.5	0.85 ± 0.16	1.28 ± 0.16	1.81 ± 0.69	2.55 ± 0.28	1.98 ± 0.15	2.13 ± 0.05
2-Hydroxy-4-(methylsulfonyl)isophthalic acid	j	70.4	9.55 ± 4.91	4.90 ± 0.39	6.44 ± 2.90	2.82 ± 0.20	1.67 ± 0.11	0.81 ± 0.02
Naringenin	i	69.8	4.91 ± 0.40	7.46 ± 0.39	7.80 ± 2.31	2.42 ± 0.53	0.89 ± 0.06	0.73 ± 0.01

*Identification letters for each peak are shown in Figure 5.

When monomers were not detected, they were labelled as n.d. RT, indicates retention time.

**TABLE 3:** GC-MS results from BHSB expressed as monomer abundance%.

Monomers	Peak*	RT	100 °C 30′	100 °C 60′	100 °C 120′	120 °C 30′	120 °C 60′	120 °C 120′
Acids								
Hexadecanoic acid	d	40.1	0.68 ± 0.23	1.05 ± 0.49	0.90 ± 0.06	0.96 ± 0.26	0.88 ± 0.17	0.69 ± 0.01
Octadecanoic acid	e	47.0	0.33 ± 0.01	0.45 ± 0.38	n.d.	0.41 ± 1.23	n.d.	n.d.
Diacids								
Hexadecanedioic acid	f	55.9	6.64 ± 0.94	5.06 ± 0.32	4.63 ± 0.49	5.62 ± 0.49	3.78 ± 0.42	4.01 ± 0.33
Hydroxy acids								
16-Hydroxyhexadecanoic acid	c	52.9	3.30 ± 1.18	4.83 ± 0.15	4.60 ± 0.28	4.55 ± 0.65	3.79 ± 1.04	4.37 ± 0.63
10,16-dihydroxyhexadecanoic acid	a	60.1	58.28 ± 1.07	58.93 ± 0.38	64.44 ± 1.17	57.39 ± 1.23	66.73 ± 4.46	67.76 ± 3.33
9,10,16-trihydroxyhexadecanoic acid	b	62.4	6.39 ± 0.37	6.37 ± 0.54	6.72 ± 0.85	6.17 ± 0.54	8.13 ± 2.16	12.59 ± 1.33
Phenolics								
4-Hydroxycinnamic acid	g	36.8	0.60 ± 0.42	1.50 ± 0.83	3.29 ± 1.11	2.59 ± 0.37	3.58 ± 2.51	2.31 ± 0.44
Resveratrol	h	64.5	0.46 ± 0.07	0.94 ± 0.06	1.69 ± 0.03	1.23 ± 0.24	1.83 ± 0.47	1.84 ± 0.04
2-Hydroxy-4-(methylsulfonyl)isophthalic acid	j	70.4	9.44 ± 3.39	5.03 ± 0.14	4.81 ± 0.28	7.39 ± 0.99	2.06 ± 0.55	0.61 ± 0.31
Naringenin	i	69.8	7.51 ± 5.28	6.07 ± 0.60	2.72 ± 0.15	3.87 ± 1.04	1.10 ± 0.26	0.68 ± 0.01

*Identification letters for each peak are shown in Figure 5.

When monomers were not detected, they were labelled as n.d. RT, indicates retention time.

The mass spectra of the 10,16OHC16, 3-O-TMS (Supplementary Figure S1 in Supplementary Material) presented a molecular weight of 504, that corresponds to the molecular weight of 1016OHC16 after the addition of three TMS groups (Si(CH_3_)_3_) from the derivatization of the sample. The major cleavages are at 331 m/z and 275 m/z, and the characteristic ions of a long-chain fatty acid of 147, 129, 103 and 95 m/z (with a reasonable concordance with the mass spectra of aleuritic acid, 4-O-TMS). The typical ion from the TMS group showed a peak at 73 m/z as reported in the literature (Nicolaides et al., 1983; Schweizer et al., 1996).

The chromatographic analysis revealed a preponderance of C16 monomers, including acids, diacids and hydroxy acids. The ω-hydroxy-acids were significantly detected (63%–85% as presented in Supplementary Tables S3 and S4), with the 9,10,16-trihydroxyhexadecanoic acid (commonly known as aleuritic acid) as the secondary major component (5%–13%). This component showed an increment with temperature and reaction time without showing a local maximum among the tested conditions. It might be possible that after 2 h of hydrolysis a fraction of the material remains as oligomers. In the case of the hexadecanedioic acid, as the only diacid detected, a decrease in abundance is observed as reaction time increases, probably due to the decarboxylation of the acids occurring under these conditions. The hexadecanoic acid presents an abundance of 1% at every temperature with the BHMW, but with the BHSB presents a lower abundance in the mildest and in the harsher conditions. From the monomers of C18 chain length, only octadecanoic acid was slightly detected (0.0%–0.7%), or not detected at all in some cases. Aromatic compounds were found in lower proportions (5%–18%) than C16 monomers and, due to their thermosensitivity, their concentration decreased with heat exposure time. These aromatics are phenolic compounds that are typically present within the cutin matrix. These findings are in concordance with previous studies on tomato peel cutin (Heredia-Guerrero et al., 2017; Pollard et al., 2008).

However, in the case of coumaric acid (4-hydroxycinnamic acid) and resveratrol, the proportion of each increase with reaction time. This observation suggests that these components may be trapped or linked within the cutin matrix, and they are released from the inside due to the depolymerization of the monomers. In contrast to the experiments conducted at 100 °C, which display a steady increase, at 120 °C this concentration decreases for coumaric acid and remains stable for resveratrol after 60 min of the reaction, presumably due to degradation caused by the elevated temperature.

Two-way ANOVA (factors: reaction time and hydrolysis method, temperature 120 °C) showed that the relative amounts of cutin hydroxy-acid monomers were significantly affected by reaction time, whereas the effect of the hydrolysis method showed significance (p > 0.05) only for the phenolics and diacids, presenting more abundance with the use of BHMW (Tables 2 and 3). It was reported in previous work the obtention of higher phenolic content compared to other methods when using microwaves in tomato industrial wastes (Solaberrieta et al., 2022). These results indicate that BHMW at 120 °C does not differentially affect the obtention of the main monomer compared to the conventional method (Supplementary Table S8). Therefore, the microwave-assisted hydrolysis can be used instead of the conventional method for the characterization of the cutin from tomato peel.

## 4 Conclusion

A comparative study between conventional heating and microwave irradiation was conducted to obtain the monomers of cutin by alkaline hydrolysis. A detailed profile of the hydrolysis products was obtained by GC-MS, with C16 hydroxy fatty acids representing the majority fraction (54.65%–67.76%) together with typical phenolic compounds characteristic of this biomass.

From a process perspective, microwave-assisted hydrolysis at 120 °C exhibited a clear advantage over the conventional method. The most significant improvement was associated with the temperature increase from 100 °C to 120 °C. This effect was confirmed under conventional alkaline hydrolysis, where the reaction time to reach a stable yield was markedly reduced from 24 h to only 1 h. On a smaller scale, the use of microwave-assisted hydrolysis further enhanced the process, leading to a 5% increase in AP yield and an additional reduction in reaction time to 40 min for achieving the maximum AP yield. Based on these results, we propose the use of microwave-assisted hydrolysis with the conditions of 60 min at 120 °C as optimal for obtention of cutin monomers.

This study provides a detailed analysis of the operating conditions and products obtained through the alkaline hydrolysis of cutin, with the aim of providing an effective process for obtaining its monomers to promote progress in the development of bio-based products and applications. Future investigations should prioritize exploring intermediate reaction conditions to enable the formation of oligomeric fractions through partial hydrolysis, which holds significant industrial potential for material applications.

## Data Availability

The original contributions presented in the study are included in the article/Supplementary Material, further inquiries can be directed to the corresponding author.

## Footnotes

1
https://www.pronat.com.es/en/home-en/
