# Supplementary material for: Enhanced microwave-assisted methodology for obtaining cutin monomers from tomato peel
Source: Front Chem. 2026 Jan 5;13:1734422. doi: 10.3389/fchem.2025.1734422 (PMC12812939; doi:10.3389/fchem.2025.1734422)
Supplement: Supplementary file 1 [file Table1.docx]

Supplementary Material

# Supplementary Figures and Tables

## Supplementary Tables

**Supplementary Table 1S**: Extractives removed from tomato peels.

| **Solvent** | **Extractives** |
| --- | --- |
| Chloroform | 3.58% ± 0.67% |
| Methanol | 1.31% ± 0.35% |
| Water | 7.98% ± 1.57% |
| Total | 12.87% ± 2.59% |

**Supplementary Table 2S:** Hydrolysis yields (Yield_SR_ and Yield_AP_) at 120°C with the BHMW method

| time (min) | Yield_SR_ | Yield_AP_ |
| --- | --- | --- |
| 0 | 86% ± 3.1% | 0% ± 0.5% |
| 10 | 51% ± 2.9% | 14% ± 0.4% |
| 20 | 33% ± 6.0% | 29% ± 2.7% |
| 30 | 20% ± 0.8% | 38% ± 2.6% |
| 60 | 15% ± 1.7% | 45% ± 1.0% |
| 90 | 10% ± 1.2% | 43% ± 4.4% |
| 120 | 10% ± 0.7% | 43% ± 2.2% |

**Supplementary Table 3S:** Hydrolysis yields (Yield_SR_ and Yield_AP_) at 120°C with the BHSB method

| time (min) | T(°C) | Yield_SR_ | Yield_AP_ |
| --- | --- | --- | --- |
| 0 | 0 | 86% ± 3.1% | 0% ± 0.5% |
| 30 | 100 | 64% ± 2.3% | 9% ± 1.0% |
| 60 | 100 | 38% ± 1.0% | 24% ± 2.9% |
| 120 | 100 | 23% ± 0.1% | 37% ± 4.0% |
| 30 | 120 | 36% ± 8.2% | 26% ± 7.3% |
| 60 | 120 | 15% ± 0.2% | 38% ± 1.3% |
| 120 | 120 | 16% ± 0.3% | 39% ± 1.1% |

**Supplementary Table 4S**: FT-IR peaks, vs= very strong, s= strong, m = medium, w= weak, vw=very weak; δ = deformation of vibration, st = stretching

|  | **Silicon Bath** | | | | | | | | | | | | **Microwaves** | | | | | | | | | | | | |  | |  | | |  | | |
| --- | --- | --- | --- | --- | --- | --- | --- | --- | --- | --- | --- | --- | --- | --- | --- | --- | --- | --- | --- | --- | --- | --- | --- | --- | --- | --- | --- | --- | --- | --- | --- | --- | --- |
|  | **100°C** | | | | | | **120°C** | | | | | | **120°C** | | | | | | | | | | | | |  | |  | | |  | | |
|  | **30'** | | **60'** | | **120'** | | **30'** | | **60'** | | **120'** | | **10 '** | | **20'** | | **30'** | | **60'** | | **90'** | | **120'** | | **RawT** | | | | |  | | |  |
|  |  |  |  |  |  |  |  |  |  |  |  |  |  |  |  |  |  |  |  |  |  |  |  |  | 3295 | | *m* | | Polysaccharides | | |  |  |
| CH_2_ st | 2925 | *vs* | 2925 | *vs* | 2925 | *vs* | 2925 | *vs* | 2924 | *vs* | 2923 | *vs* | 2925 | *vs* | 2925 | *vs* | 2925 | *vs* | 2923 | *vs* | 2925 | *vs* | 2923 | *vs* | 2923 | | *m* | | Cutin, aliphatic | | |  |  |
| CH_2_ st | 2851 | *s* | 2853 | *s* | 2852 | *s* | 2851 | *s* | 2851 | *vs* | 2850 | *vs* | 2852 | *s* | 2852 | *s* | 2852 | *s* | 2852 | *s* | 2852 | *s* | 2850 | *s* | 2852 | | *m* | | Cutin, aliphatic | | |  |  |
| ester |  |  | - |  | - |  |  |  | - |  | - |  | - |  | - |  | - |  | - |  | - |  | - |  | 1730 | | *m* | | Cutin, esters | | |  |  |
| acid | 1703 | *s* | 1705 | *s* | 1705 | *vs* | 1705 | *vs* | 1702 | *vs* | 1701 | *vs* | 1705 | *s* | 1703 | *s* | 1705 | *vs* | 1703 | *vs* | 1703 | *vs* | 1701 | *vs* | - | |  | | Cutin, acids | | |  |  |
| acid | 1634 | *vs* | 1636 | *m* | 1636 | *m* | 1636 | *m* | 1637 | *w* | 1637 | *w* | 1634 | *m* | 1632 | *m* | 1634 | *w* | 1634 | *w* | 1634 | *w* | 1638 | *w* | 1626 | | *m* | | Phenolic compounds | | |  |  |
| aromatic | - |  | 1604 | *m* | 1604 | *m* | - |  | 1604 | *w* | 1604 | *w* | 1605 | *m* | 1605 | *m* | 1605 | *w* | 1605 | *w* | 1603 | *w* | 1605 | *w* | 1605 | | *m* | | Phenolic compounds | | |  |  |
| aromatic | 1515 | *m* | 1515 | *w* | 1515 | *w* | 1515 | *w* | 1516 | *vw* | 1515 | *vw* | 1516 | *w* | 1516 | *w* | 1516 | *w* | 1516 | *w* | 1516 | *w* | 1516 | *vw* | 1514 | | *w* | | Phenolic compounds | | |  |  |
| CH_2_ st | 1454 | *m* | 1459 | *m* | 1463 | *m* | 1460 | *m* | 1463 | *w* | 1464 | *w* | 1461 | *m* | 1461 | *m* | 1463 | *m* | 1463 | *m* | 1463 | *m* | 1463 | *m* | 1452 | | *w* | | Cutin, aliphatic | | |  |  |
| aromatic | 1409 | *m* | 1411 | *w* | 1411 | *w* | 1411 | *w* | 1412 | *w* | 1411 | *m* | 1410 | *w* | 1410 | *w* | 1412 | *w* | 1410 | *w* | 1410 | *w* | 1410 | *m* | - | |  | | Phenolic compounds | | |  |  |
| C-O st | 1340 | *m* | 1340 | *m* | 1344 | *w* | 1348 | *w* | 1346 | *w* | 1348 | *w* | 1342 | *m* | 1342 | *m* | 1344 | *w* | 1344 | *w* | 1346 | *w* | 1350 | *w* | - | |  | | Cutin, esters | | |  |  |
| OH |  |  | - |  | - |  |  |  | - |  | - |  | - |  | - |  | - |  | - |  | - |  | - |  | 1236 | | *m* | | Cutin, polysaccharides | | |  |  |
| O-H δ | 1219 | *s* | 1217 | *s* | 1217 | *s* | 1219 | *m* | 1222 | *m* | 1222 | *m* | 1218 | *m* | 1218 | *m* | 1222 | *s* | 1220 | *m* | 1218 | *m* | 1218 | *m* | - | |  | |  | | |  |  |
| ester | 1163 | *s* | 1167 | *s* | 1167 | *s* | 1167 | *s* | 1168 | *m* | 1169 | *m* | 1167 | *s* | 1165 | *s* | 1167 | *s* | 1167 | *m* | 1167 | *m* | 1167 | *m* | 1161 | | *m* | | Cutin, esters | | |  |  |
| ester | 1106 | *m* | 1107 | *w* | 1107 | *w* | 1106 | *m* | 1107 | *w* | 1118 | *w* | 1106 | *m* | 1106 | *m* | 1106 | *m* | 1106 | *w* | 1108 | *w* | 1110 | *w* | 1100 | | *s* | | Cutin, esters | | |  |  |
| ester | 1054 | *m* | 1056 | *w* | 1057 | *w* | 1054 | *m* | 1057 | *w* | 1058 | *w* | 1051 | *s* | 1051 | *s* | 1047 | *s* | 1051 | *m* | 1049 | *m* | 1053 | *m* | 1053 | | *vs* | | Polysaccharides | | |  |  |
|  | - |  | - |  | - |  | - |  | 1042 | *w* | 1041 | *w* | - |  | - |  | - |  | - |  | - |  | - |  | 1022 | | *vs* | | Polysaccharides | | |  |  |
| C-O st | 981 | *w* | 983 | *w* | 982 | *w* | 981 | *w* | 980 | *w* | 981 | *w* | 979 | *w* | 979 | *w* | 979 | *m* | 981 | *w* | 983 | *w* | 979 | *w* | - | |  | | Phenolic compounds | | |  |  |
| aromatic | 834 | *w* | 834 | *w* | 834 | *w* | 834 | *w* | 835 | *w* | 835 | *vw* | 834 | *w* | 834 | *w* | 834 | *w* | 834 | *w* | 834 | *w* | 836 | *vw* | 832 | | *vw* | | Phenolic compounds | | |  |  |

**Supplementary Table 5S**: main families of monomers expressed in relative abundance from the BHMW.

**Supplementary Table 6S**: main families of monomers expressed in relative abundance from the BHSB.

**Supplementary Table 7S:** Relative areas and standard deviation of main monomer from Figure 6

| T(°C) | time (min) | Rel. Area | T(°C) | time (min) | Rel. Area |
| --- | --- | --- | --- | --- | --- |
| 120 | 10 | 54.65 ± 2.56 | 100 | 30 | 58.28 ± 1.07 |
| 120 | 20 | 57.02 ± 0.84 | 100 | 60 | 58.93 ± 0.38 |
| 120 | 30 | 60.01 ± 3.97 | 100 | 120 | 64.44 ± 1.17 |
| 120 | 60 | 64.25 ± 2.38 | 120 | 30 | 57.39 ± 1.23 |
| 120 | 90 | 67.73 ± 2.36 | 120 | 60 | 66.73 ± 2.46 |
| 120 | 120 | 65.76 ± 1.33 | 120 | 120 | 67.76 ± 3.33 |

**Supplementary Table 8S:** Statistical differences between BHMW and BHSB at 120°C and 30, 60 and 120 minutes of reaction time. In the column of p-value the ones that present significant differences (p < 0.05) are shown with *. For octadecanoic acid it was not possible to estimate differences due to the lack of its presence in several conditions.

| Compound | Time (min) | BHMW ± SD | BHSB ± SD | P-value |
| --- | --- | --- | --- | --- |
| 10,16-dihydroxyhexadecanoic acid | 30 | 60.01 ± 3.97 | 57.39 ± 1.23 | 0.3364 |
|  | 60 | 64.25 ± 2.38 | 66.73 ± 4.46 | 0.4344 |
|  | 120 | 65.76 ± 1.33 | 67.76 ± 3.33 | 0.3888 |
| 9,10,16-trihydroxyhexadecanoic acid | 30 | 6.68 ± 0.69 | 6.17 ± 0.54 | 0.6409 |
|  | 60 | 8.50 ± 0.26 | 8.13 ± 2.16 | 0.8355 |
|  | 120 | 10.25 ± 0.92 | 12.59 ± 1.33 | 0.0546 |
| 16-hexadecanoic acid | 30 | 3.97 ± 1.14 | 4.55 ± 0.65 | 0.7993 |
|  | 60 | 3.34 ± 0.74 | 3.79 ± 1.04 | 0.6464 |
|  | 120 | 3.65 ± 0.02 | 4.37 ± 0.63 | 0.1517 |
| hexadecanedioic acid | 30 | 4.22 ± 0.58 | 5.62 ± 0.49 | 0.3281 |
|  | 60 | 3.84 ± 0.28 | 3.78 ± 0.42 | 0.8615 |
|  | 120 | 2.45 ± 0.07 | 4.01 ± 0.33 | 0.0014 * |
| hexadecanoic acid | 30 | 0.85 ± 0.14 | 0.96 ± 0.26 | 0.5735 |
|  | 60 | 0.83 ± 0.11 | 0.88 ± 0.17 | 0.7612 |
|  | 120 | 0.84 ± 0.03 | 0.69 ± 0.01 | 0.0011 * |
| 4-hydroxycinnamic acid | 30 | 1.43 ± 0.58 | 2.59 ± 0.37 | 0.0436 * |
|  | 60 | 5.04 ± 0.45 | 3.58 ± 2.51 | 0.0024 * |
|  | 120 | 3.86 ± 1.03 | 2.31 ± 0.44 | 0.0750 |
| resveratrol | 30 | 1.81 ± 0.69 | 1.23 ± 0.24 | 0.2452 |
|  | 60 | 2.54 ± 0.28 | 1.83 ± 0.47 | 0.1549 |
|  | 120 | 2.13 ± 0.05 | 1.84 ± 0.04 | 0.0017 * |
| naringenin | 30 | 7.80 ± 2.31 | 3.87 ± 1.04 | 0.0552 |
|  | 60 | 2.42 ± 0.53 | 1.10 ± 0.26 | 0.0312 * |
|  | 120 | 0.72 ± 0.01 | 0.69 ± 0.01 | 0.0299 * |
| 2-hydroxy-4-(methylsulfonyl)isophthalic acid | 30 | 6.44 ± 2.90 | 7.39 ± 0.99 | 0.6199 |
|  | 60 | 2.82 ± 0.20 | 2.06 ± 0.55 | 0.1728 |
|  | 120 | 0.81 ± 0.02 | 0.61 ± 0.31 | 0.9850 |

## Supplementary Figures


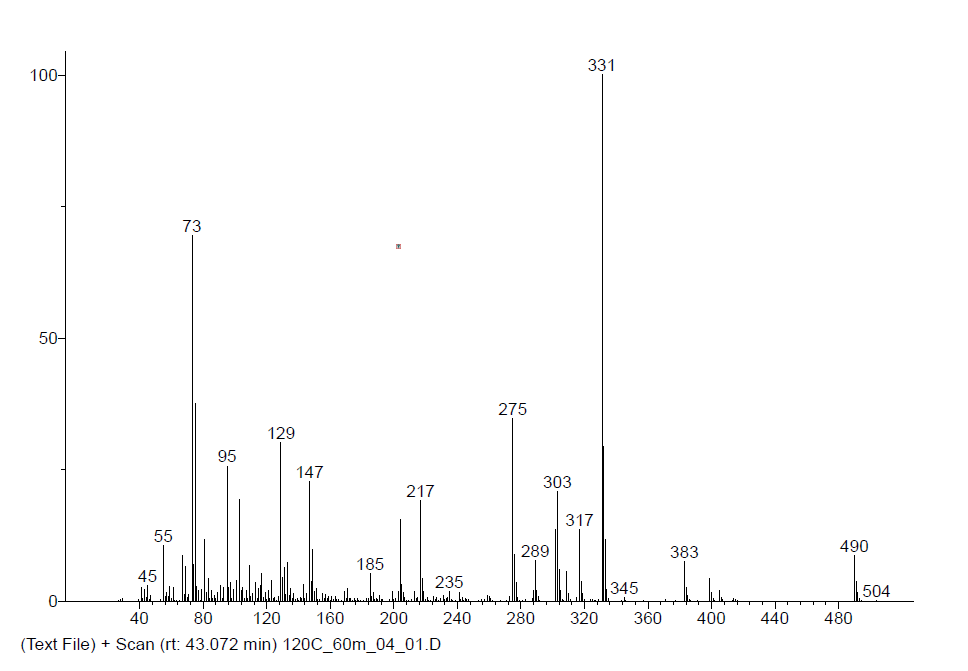


**Supplementary Figure 1S:** Mass spectra m/z of the derivatized main monomer from cutin
